# Anthropogenic nitrate in groundwater and its health risks in the view of background concentration in a semi arid area of Rajasthan, India

**DOI:** 10.1038/s41598-021-88600-1

**Published:** 2021-04-29

**Authors:** Abdur Rahman, N. C. Mondal, K. K. Tiwari

**Affiliations:** 1 Earth Process Modeling Group, CSIR-National Geophysical Research Institute, Hyderabad, India; 2 Academy of Scientific & Innovative Research (AcSIR), Ghaziabad, 201002 India; 3 National Institute of Technology (NIT), Garhwal, Uttarakhand India

**Keywords:** Environmental sciences, Hydrology, Health care, Risk factors

## Abstract

An increased nitrate (NO_3_^−^) concentration in groundwater has been a rising issue on a global scale in recent years. Different consumption mechanisms clearly illustrate the adverse effects on human health. The goal of this present study is to assess the natural and anthropogenic NO_3_^−^ concentrations in groundwater in a semi arid area of Rajasthan and its related risks to human health in the different groups of ages such as children, males, and females. We have found that most of the samples (n = 90) were influenced by anthropogenic activities. The background level of NO_3_^−^ had been estimated as 7.2 mg/L using a probabilistic approach. About 93% of nitrate samples exceeded the background limit, while 28% of the samples were beyond the permissible limit of 45 mg/L as per the BIS limits. The results show that the oral exposure of nitrate was very high as compare to dermal contact. With regard to the non-carcinogenic health risk, the total Hazard Index (HI_Total_) values of groundwater nitrate were an average of 0.895 for males, 1.058 for females, and 1.214 for children. The nitrate health risk assessment shows that about 38%, 46%, and 49% of the samples constitute the non-carcinogenic health risk to males, females, and children, respectively. Children were found to be more prone to health risks due to the potential exposure to groundwater nitrate.

## Introduction

Groundwater, especially in arid and semi arid regions, is typically the most valuable water resource, and so the conservation of groundwater supplies is important worldwide^1^. However, over the past few decades, groundwater quality in most aquifers of the world has declined due to increased human effects^2^. In this modern era, rapid development, population explosion, urbanisation, industrialization, tremendous use of fertilisers in irrigated fields, improper sewage systems, human and animal waste contribute to groundwater pollution^3^. Groundwater pollution is closely correlated with diffuse (non-point) sources for agricultural operations. In most agricultural practices several types of inorganic and organic fertilisers are used^4–6^. The risk of groundwater pollution is raised by the improper use of chemicals and fertilisers. Once the groundwater is polluted, remediation is difficult. Thus, the prevention of pollution is also the key water quality control policy^7^. The primary source of inorganic nitrogen within the soil is nitrate, which is necessary for healthy growth and development of crops^8^.

Nitrate (NO_3_^−^) is well-known environmental pollutant that not only arises naturally, but also is released by a number of anthropogenic exercises. These anthropogenic activities include the manufacture and use of nitrate fertilizers, fossil fuel combustion (occur as atmospheric deposition, hereinafter AD) and releases of both domestic and industrial sewage systems and modification in nitrogen-fixing crops in natural vegetation^9–11^. In most natural waters, nitrate forms a critical portion of the ionic charge. Because of the harmful effects on humans at high doses, NO_3_^−^ ions are used in international regulations and guidelines^12^. However, the long-term intake of elevated nitrate concentrations can cause serious health hazards in children, such as methemoglobinemia, which is also known as a blue baby syndrome, and stomach cancer in adults as well^13,14^. In view of this, the World Health Organisation (WHO) has defined the maximum nitrate level of a contaminant in drinking water as 50 mg/L. As per the Indian situation, 45 mg/L is recommended by the Bureau of Indian Standards (BIS) as the permissible level of NO_3_^−^ in drinking water. As a result of the prolonged ingestion of groundwater nitrate, serious health issues are encountered in the different parts of the world. Thus, nitrate exposure-related health risk assessment research is extensively studied in different countries such as China^15–18^, Pakistan^19^, Iran^20,21^, Mexico^22^ and India^3,23–25^.

The USGS in 1994 defines background concentration as ‘‘a concentration of a substance in a particular environment that is indicative of minimal influence by human (anthropogenic) sources’’. Usually, background levels are estimated either temporally (concentrations before anthropogenic activity) or spatially (concentrations in the areas not influenced by anthropogenic activity)^26^. It may be difficult to establish background concentrations for certain pollutants, mainly those that have several non-point sources or environmentally reactive. Nitrate (NO_3_^−^) is an example of  anion for which, it is difficult to establish a threshold concentration due to its different geogenic and anthropogenic origins and its reaction^26,27^. The issues with establishing a nitrate (NO_3_^−^) threshold value, is that natural or geogenic processes will differ greatly in time and space that influences NO_3_-N concentrations. It is generally found that NO_3_-N concentrations in aquifers decrease with down gradation or with depth^26,28^. If the groundwater reaches at redoxcline depth (redox boundary), where nitrate and oxygen suddenly vanish due to denitrification, this will occur within a relatively slight difference in depth^29,30^. These redox boundaries can shift with time, and there may be a limit to how much NO_3_^−^ can be reduced in some aquifers, mainly those with low concentration organic carbon and ferric iron^31^. Thus an important aspect influencing the determination of background concentrations is the well depth from which groundwater samples are taken. The NO_3_-N concentrations can also be influenced by the water mixing of different ages and/or from different origin. Mixing can take place naturally (e.g., due to quick recharge) or anthropogenically (e.g., due to construction of the well, wells are frequently uncased over greater depth). Other significant variables include lithology, soil composition, the thickness of unsaturated zone, and bioactivities^26^.

The investigated area falls under the arid and semi arid climate of Rajasthan where the only source of fresh water is groundwater. According to the CGWB report^32^, most of the areas of the state have been categorised as over-exploitation zones. Along with the over-exploitation of groundwater, the quality of this precious resource is also highly degraded. But it has been seen that nobody discussed the details about the nitrate contamination in a regional scale including its natural background which is a crucial parameter to know about anthropogenic influences except a general assessment of the groundwater quality^33^. Therefore, the main objectives of this research are: to (1) analyse groundwater chemistry, (2) evaluate regional natural background concentration of nitrate, (3) investigate nitrate toxicity for understanding the potential source and contamination mechanism of nitrate, and (4) quantify the potential non-carcinogenic health risks induced by groundwater exposure to nitrate using recommended model of the US Environmental Protection Agency^34,35^. It will provide an important scientific and logical understanding of the human health risks of NO_3_^−^ in groundwater and also help to improve the groundwater quality.

## Material and Methods

### Study area

**Figure 1:**
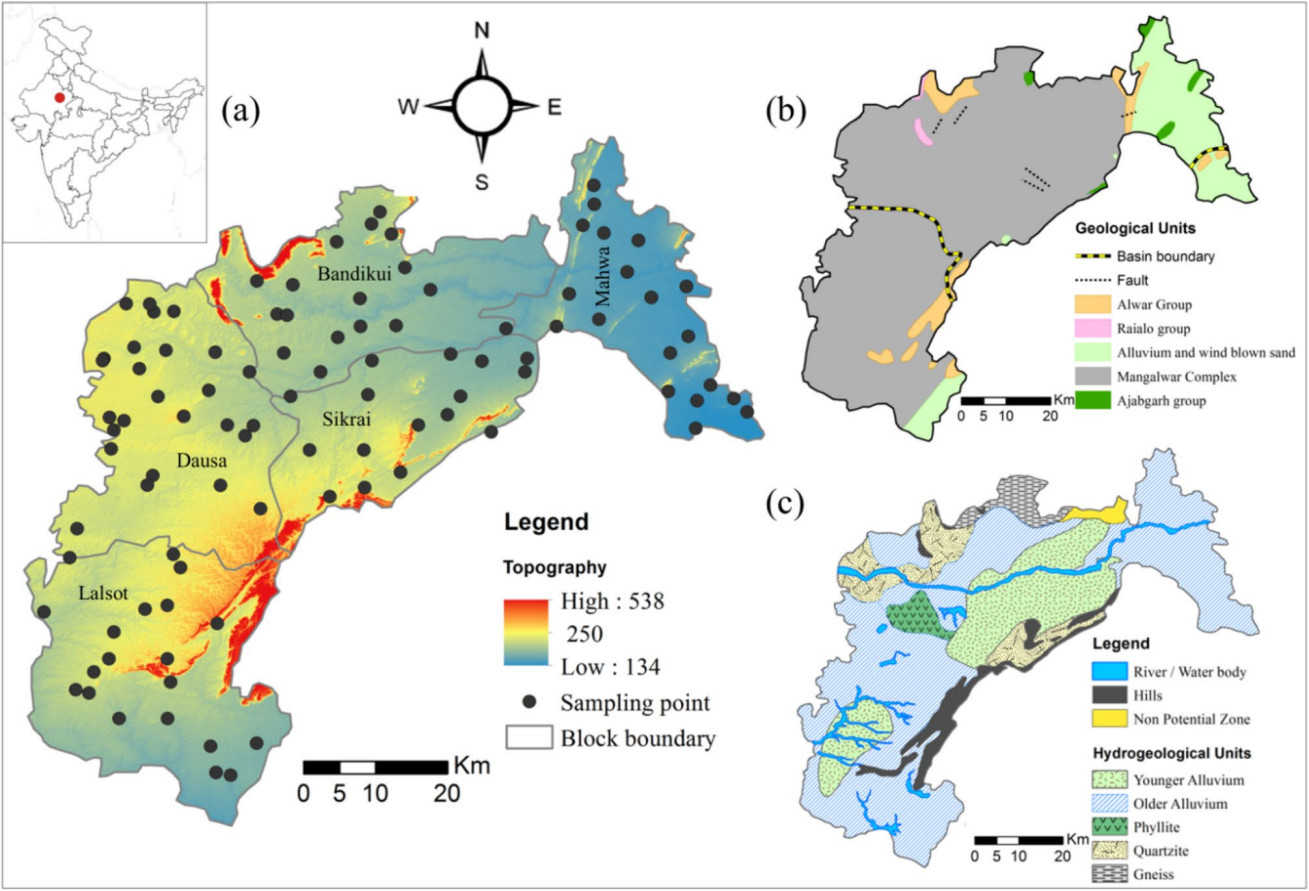
Showing the maps of (**a**) Topography in meters above mean sea level and the sampling location of Dausa district, Rajasthan, India, (**b**) regional geology, and (**c**) hydrogeology with the potential aquifers. (ArcGIS Desktop. 10.3. ESRI, California, US. https://desktop.arcgis.com).

The area falls between 26° 22′ 13.32″ to 27° 14′ 33.58″ north latitudes and 76° 08′ 32.62″ to 77° 05′ 00.41″ east longitudes, covering an area of about 3420 km^2^ (Fig. 1a). Three significant river basins are present in the study area; Banganga basin (covering about 63% of the study area in the northern part), Morel basin (covering about 34% of area in the southern part), and a small part by Ghambhir basin (around 3% of the area in the south of Mahwa)^36^. According to the census 2011, the population in the study area was ~ 1.03 million with the population density of 476 people per square kilometre. Most of the area is covered by agricultural land in which agriculture production is scattered over both kharif, and rabi cultivation; kharif cultivation is based on precipitation (rainfed) and rabi cultivation is especially based on groundwater source.

Because of significant daily temperature variations and low, moderate rainfall, the climate of the area can be categorized as semi arid. It is markedly seasonal and is characterised by a dry and steadily increasing hot season between March and May, a dry and cold winter between October and February, but from June to September a monsoon season. In the study region, the minimum and maximum temperatures reported are below 10 °C in January, and 48 °C in June. The gross annual evapotranspiration potential is 1744.7 mm^32^.

### Geology and hydrogeology

In the study area, the Alwar group of rocks consists of quartzite and schist, alluvium and wind-blown sand occupied the north-eastern and south-western parts (Fig. 1b). The 3/4^th^ portion of the study area is covered by quaternary alluvium (Younger alluvium: ~ 21.6%, and older alluvium: ~ 58.4%) consisting of Aeolian and fluvial silt, sand, gravel, and occasional pebbles^36^. In hard rock settings, the weathered, fractured, and jointed hard rocks form aquifers. Quartzite contributes to approximately 9.0% of the aquifer, gneisses and phyllite aquifers occupy 5.3% of the remaining Quaternary alluvium is the primary water-bearing formation covering about 84.65% of the area^32^. Talus and scree deposits and hard rocks of Bhilwara and Delhi Super Group rest in a small portion forming about 15.35% of the minor aquifers. Alluvium sediments including sand, kankar (calcareous detrital materials), clay, and gravel form the potential aquifer in the study area (Fig. 1c). The occurrence of groundwater is marked under unconfined to confined aquifer conditions with the primary porosity of alluvium^33^. The geological and hydrogeological map (Fig. 1b and Fig. 1c) were made by ArcGIS Desktop. 10.3. ESRI, California, US. (https://desktop.arcgis.com) after Ground Water Department, Rajasthan (https://energy.rajasthan.gov.in).

### The data used for this study

Hydrochemical data were collected and analysed by the State Groundwater Department Jaipur, Rajasthan. These samples were collected in 1.0 L polyethylene bottles and used for the analysis of major ions including nitrate. Before collecting groundwater samples, the wells were drained for a sufficient time so that the accumulated water, if any, was absolutely removed from storage within the assembly of the well. As defined by the American Public Health Association^37^, the ions were analysed by the standard methods. The portable kits such as electrical conductivity (EC) and pH meters were used to measure the EC and pH at the time of sampling. Major ions including Na^+^ and K^+^ were analysed by flame photometer, NO_3_^−^ and F^−^ by UV–visible spectrophotometer and remaining ions and total hardness (TH) by volumetric methods at Regional Chemical Laboratory, Ground Water Department, Jaipur, Rajasthan. To ensure the accuracy of the analytical results, laboratory quality-assurance (QA) and quality-control (QC) methods were used, including standard calibration and standard operating procedures. Nitrate datasets of past eight years (2011–2018) had been analysed to see the trend of change in the concentration. But the data in the year 2018 was extensively utilised to assess anthropogenic nitrate in groundwater and its health risks. After validation of reliability of data, about 97 samples, out of 101 collected data, were used for background analysis and hydrochemical characterization.

### Charge balance error

In order to verify the validity and reliability of the analysed hydrochemical dataset, the cation–anion balance procedure was carried out. The basic law of electro-neutrality allows the number of cations to be equal to or almost equal to the number of anions. The error was measured using the methods implemented^38^ to validate and remove low-quality samples. The ion concentration unit was first changed from milligram per litre (mg/L) to milliequivalent per litre (meq/L). To calculate the ionic balance error of each sample, the following given formula was used^39,40^.

1 $$Charge\;balance\;error=\left\{ {\frac{(\sum Cations -\sum Anions)}{{(\sum Cations + \sum Anions)}}} \right\} \times100$$

The calculation of groundwater quality results may be good if the charge balance error is <  ± 5%, and if the charge balance error is > ± 5%, it will be considered as poor^41^. However, the charge balance error of up to ± 10% is acceptable for groundwater^42^. The value would not pass the validation test if it is greater than ± 10. Among the 101 collected samples, 4 samples had been excluded due to an error of more than ± 10%. About 97 samples, out of 101, fell within the 10% charge balance errors, which were considered to be reliable in this research work.

### Natural background

Anthropogenic additions above background concentration from the numerous sources of nitrate are now a global issue. Additionally, it can be difficult to determine the background concentration of a specific chemical component. The criteria for identification of anthropogenic contamination had been adopted by using the probability distribution of random datasets. As a brief way of picking separate population as events in hydrogeochemical databases of a given chemical species, the cumulative probability (CP) approach has been introduced^43^. This method is quick and convenient for identifying the different population with the help of inflection point^26,44–47^ without any type of pre-selection of data. In the lack of long-term temporal databases, this method gives more reliable outcomes^47^. Shapiro–Wilk test^48^ was used to test the normality assumption of the different populations of the datasets. The SPSS statistics package programme was utilised to do all the statistical analysis (SPSS software, IBM SPSS product version 25.0 at P < 0.05). A value of P < 0.05 had been measured as statistically significant in all data analyses.

### Health risk assessment

In general, the intake of polluted groundwater can cause a severe threat to humans, primarily by two exposure routes, first one is the ingestion of drinking water or oral route, and the second one is the dermal interaction route^23,49^. The US Environmental Protection Agency originally proposed this rigorous model for the assessment of human health risk^34,35^. In this study, the risk assessment was carried out in three groups of the exposed population, comprising children, females, and males.

The non-carcinogenic health risk from oral intake was calculated as follows^3,15,16,50^.

2 $$CDI = \frac{C \times EF \times ED \times IR}{{ABW \times AET}}$$

3 $$HQ_{Oral} = \frac{CDI}{{RfD}}$$

where, in Eq. (2), CDI is referred as chronic daily intake (in mg/kg/day); “C” is the concentration of groundwater nitrate (in mg/L); IR is denoted for daily ingestion rate of groundwater (in L/day) for both males and females ingestion rate is 2.5 L/day and for children, ingestion rate is 1 L/day ^34^ EF is denoted for the exposure frequency (in days/year), and the exposure frequency is considered as 365 days/year for males, females, and children^34^, ED is denoted for exposure duration (in a year), for children 12 years, for females 67 years, and for males, 64 years have considered for this study^23,25^. ABW is the average body weight as 65 kg, 55 kg, and 15 kg for males, females, and children, respectively^3^. The average exposure times (AET) are 23,360 days, 24,455 days, and 4380 days for males, females, and children, respectively. In Eq. (3), the hazard quotient is presented as HQ. RfD indicates reference dose of nitrate contaminant (in mg/kg/day) which is 1.6 mg/kg/day^34,35^.

The non-carcinogenic health risk from dermal contact is calculated by the following formulae^3,15,16,50^.

4 $$DAD = \frac{{C \times TC \times K_{i} \times CF \times EV \times ED \times EF \times SSA }}{ABW \times AET}$$

5 $$HQ_{Dermal} = \frac{DAD}{{RfD}}$$

6 $$HI_{Total} = \mathop \sum \limits_{i = 1}^{n} \left( {HQ_{Oral} + HQ_{Dermal} } \right)$$

where in Eq. (4) DAD indicates the dermal absorbed dose (in mg/kg × day); TC is the contact time (in h/day) taken as 0.4 in h/day; Ki represents the dermal adsorption parameters (in cm/h) taken as 0.001 cm/h; and CF is denoted for conversion factor taken as 0.001^34,51^. EV represents bathing frequency (in times/day) and considered as one time in a day, and SSA indicates the skin surface area (in cm^2^) and values for SSA are taken as 16,600 sq. centimetres for both males and females, and 12,000 sq. centimetres for children^35,51^. In Eq. (6), HI is the hazard index, and non-carcinogenic human health risk is denoted by the value of HI. The HI value greater than one shows the potential human health risk from nitrate contamination, and HI value less than one expresses an acceptable level of health risk on human^35,51^.

### Data treatment

Descriptive statistics were carried out using IBM SPSS product version 25.0 (https://www.ibm.com), and the sample concentration results were compared with the Indian Standard of drinking water quality^52^. We performed principal component analysis (PCA), a commonly used statistical tool for extracting critical knowledge from multivariate data, based on the correlation coefficients^53,54^. ArcGIS 10.3 software (ESRI, Redlands, California, USA, https://desktop.arcgis.com) and Surfer 13 (https://www.goldensoftware.com) were used to make distribution maps and interpolate the experimental dataset.

## Results and discussion

### Hydrochemical examination

The detailed statistics of hydrochemical parameters and their drinking limits suggested by the Bureau of Indian Standards^52^ for groundwater quality are presented in Table 1. The electrical conductivity (EC) values of groundwater varied between 290 and 6300 µS/cm, with the mean value of 1712.5 µS/cm. The pH value ranged from 8.1 to 9.6, with a mean value of 8.7, indicating the groundwater is alkaline in nature. An important characteristic factor, presenting dissolved chemical concentrations, was the total dissolved solids (TDS) values. The TDS value varied between 158.4 mg/L and 3826.4, with the mean value of 998.3 mg/L. The hydrochemical analysis indicates that about 71% of the samples had been exceeded the drinking limits of the BIS guidelines^52^ in the context of the TDS values. Among cations, Na^+^ was observed predominant, followed by Mg^2+^ > Ca^2+^ > K^+^ based on average cation concentrations. Among anions, HCO_3_^−^ was observed as the predominant anion, followed by Cl^−^ > SO_4_^2−^ > CO_3_^−^ > NO_3_^−^ > F^−^. Bicarbonate was the key component of all largely dissolved groundwater ions in the study area. High concentrations of HCO_3_^−^ were found and ranged from 48.8 to 951.9 mg/L, with the mean value of 329.6 mg/L. The concentrations of Na^+^ , K^+^ , Ca^2+^, Mg^2+^, SO_4_^2−^ and F^−^ ranged from 15.2 to 1287.4, 0.8 to 140.8, 2.0 to 62.1, 6.1 to 175.1, 19.2 to 754.1, and 0.2 to 4.8 respectively. The elevated concentration of Cl^−^ was observed, and it ranges 21.3 to 1574.0 mg/L, with an average of 268.0 mg/L. The spatial distribution of hydrochemical parameters has shown in Fig. S1. The elevated level of Cl^−^ was noticed in the northern central, and southern parts of the study area (Fig. S1). The northern portion and the western side of the study area were the zones where EC was high in the groundwater (Fig. S1). The elevated concentrations of SO_4_^2−^ were noticed in the western and north eastern part of the study area while F^−^ was high in southern and central part (Fig. S1). The result indicates around 28% of groundwater samples of NO_3_^−^ were beyond the BIS limit, however, the elevated concentrations of NO_3_^−^ were located especially in the northern, central and southern part of the study area. The heterogeneous distribution of the hydrochemical variables in groundwater also indicates the discrepancy in the coefficient of variance (CV%) values^3,54^. The CV of several hydrochemical parameters (K^+^, SO_4_^2−^, NO_3_^−^, Cl^−^, and F^−^) in groundwater is more than 100 percent in terms of variance coefficient (CV), as shown in Table 1. Very High CV of K^+^ (527.19%), SO_4_^2−^ (204.95%), Cl^−^ (189.70%) and NO_3_^−^ (149.30%) demonstrate the potential pollution by anthropogenic activities in the study area.

**Table 1:** Statistics of groundwater quality parameters and the exceeding percentage of each parameter based on the BIS guidelines (2012).

Parameters	Min	Max	Mean	SD	Median	CV %	BIS Standard, 2012	Sample exceeding BIS guidelines (%)
pH	8.1	9.6	8.7	0.28	8.8	3.20	6.5–8.5	78
EC	290.0	6300.0	1712.5	1258.32	1340.0	93.90	1500	47
TDS	158.4	3826.4	998.3	760.41	812.6	93.57	500	71
Na^+^	15.2	1287.4	297.4	255.91	223.0	114.76	200	57
K^+^	0.8	140.8	8.8	20.61	3.9	527.19		
Ca^2+^	2.0	62.1	14.9	10.59	12.0	88.09	75	
Mg^2+^	6.1	175.1	46.3	33.79	36.5	92.62	30	65
Cl^−^	21.3	1574.0	268.0	309.34	163.1	189.70	250	35
SO_4_^2−^	19.2	754.1	145.4	177.19	86.5	204.95	200	13
CO_3_^−^	12.0	144.2	59.5	32.32	57.1	56.60		
HCO_3_^−^	48.8	951.9	329.6	174.27	305.1	57.12	200	75
NO_3_^−^	1.2	161.8	37.2	37.03	24.8	149.30	45	28
F^−^	0.2	4.8	1.0	0.82	0.8	102.92	1	33
TH	80.0	855.0	227.7	145.99	195.0	74.87	200	42

EC is Electrical Conductivity (µS/cm = micro Siemens per centimetre) at 25 °C, TDS is Total Dissolved Solids, SD is standard deviation, CV is coefficient of variance, TH is Total Hardness, All are in mg/L except pH and EC.

### Background level of nitrate

**Figure 2:**
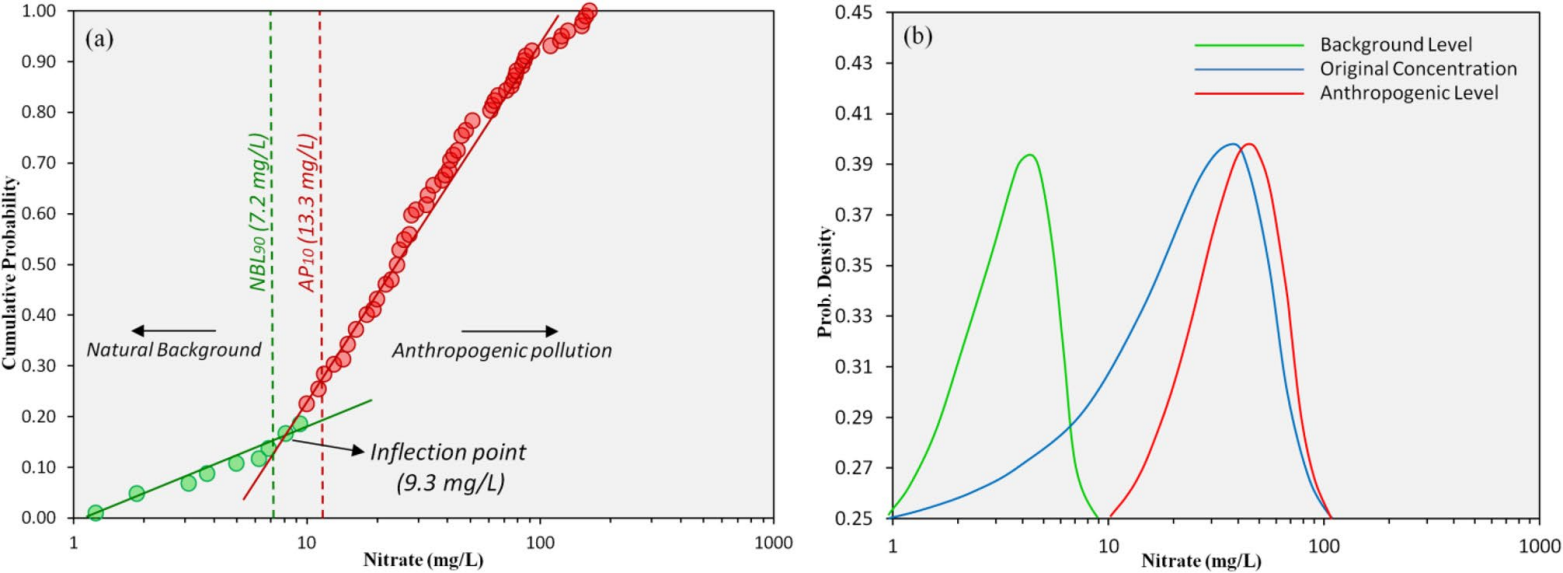
Showing (**a**) Cumulative probability plots of the NO_3_^−^ concentration in groundwater on which two normal subpopulations events (estimated NBL; natural background level, and APL; anthropogenic level). In each plot, a single inflection point is observed on log-scale, and (**b**) Probability density curve of a distinct population of NO_3_^−^ concentration in groundwaters.

Most published NO_3_^−^ concentrations have been described as anthropogenic groundwater contamination, which were estimated by different techniques. In this study the background concentration of nitrate in groundwater had been estimated using a statistical approach (mainly the cumulative probability distribution of the dataset). The cumulative probability method was used to differentiate between various nitrate populations occurrences in groundwater. The background concentrations and anthropogenic concentrations of nitrate had been marked with a sharp inflection point at 9.3 mg/L (Fig. 2a). Then the natural background level (NBL) and anthropogenic level (APL) were calculated as the 90^th^ percentile (7.2 mg/L) and 10^th^ percentile (13.3 mg/L) of the events separated by inflection point, respectively. Probability density curves with respect to the nitrate concentration of distinct populations (NBL, APL, and original) have been shown in Fig. 2b.

The Shapiro–Wilk test was used (in Table S1) to test the normality of different populations (NBL and APL). If the Shapiro–Wilk test significance value is greater than 0.05, the data is normal. If it is smaller than 0.05, the data will deviate greatly from the normal distribution. In this test, the significance value of NBL was obtained as 0.072, which indicated the normality of NBL, while the significance value of APL and original concentrations of nitrate indicated abnormality. Thus this test strengthens and supports the background value of nitrate in the groundwater of the study area. The background concentration of nitrate was high compared to other works related to the nitrate background^26^. This high level of background concentration might have been affected by anthropogenic factors^46^. However, the spatial distribution of NBL along total concentration of nitrate illustrates that the variation between NBL and the experimental concentration of NO_3_^−^ was high in the northern, central, and southern parts of the study area. The same areas were marked as potential health risk threats for adults and children.

### Nitrate distribution and mechanism of contamination

Groundwater nitrate vulnerability can be described as the sensitivity of an aquifer's susceptibility to nitrate contamination, based on the redox environment in both the aquifer material and the overlying geological strata^55,56^. In groundwater, the presence of nitrate is due to various sources and origins. Mixing of water from various sources may affect NO_3_^−^ concentrations and is a frequent occurrence in unconfined and fractured aquifers. Mixing of water can occur naturally (e.g., due to rapid recharge) or due to the poor well construction activities. These variables can change both inside and across groundwater basins or watersheds. Nitrate contamination occurs in several environments (for example in groundwater and surface water including lake, river, etc.**)** for different causes, viz. septic tank leakage, fertilisers used in farm fields, landfill leachate, leakage of sewerage pipes, drainage of poultry waste, and household / cultivation of animal dung^50,57–59^. There are many approaches that can apply to the overall nitrate concentration of ordinary groundwater, such as geological characteristics, environments, anthropogenic activities, nitrogen-bearing soil, and atmospheric nitrogen fixation^60^. The disparity in NO_3_^−^ content in various inspection areas may be due to cyclical precipitation, the energy rate of subsurface water, mechanism of evapotranspiration, and so on^61–63^. Nitrate is usually quickly dissolved in surface water and readily enters the groundwater as well. In addition, an oxidising state in the landfill site may also cause a nitrification reaction that normally converts ammonia (NH_3_) to nitrate (NO_3_^−^), and hence also raises nitrate concentration in the groundwater^3,22,64,65^. The following equation can be understood as the whole nitrification mechanism^66^.

7 $${\text{NH}}_{4} + 2{\text{O}}_{2} \to {\text{NO}}_{3}^{ - } + {\text{H}}_{2} {\text{O}} + 2{\text{H}}^{ + }$$

**Figure 3:**
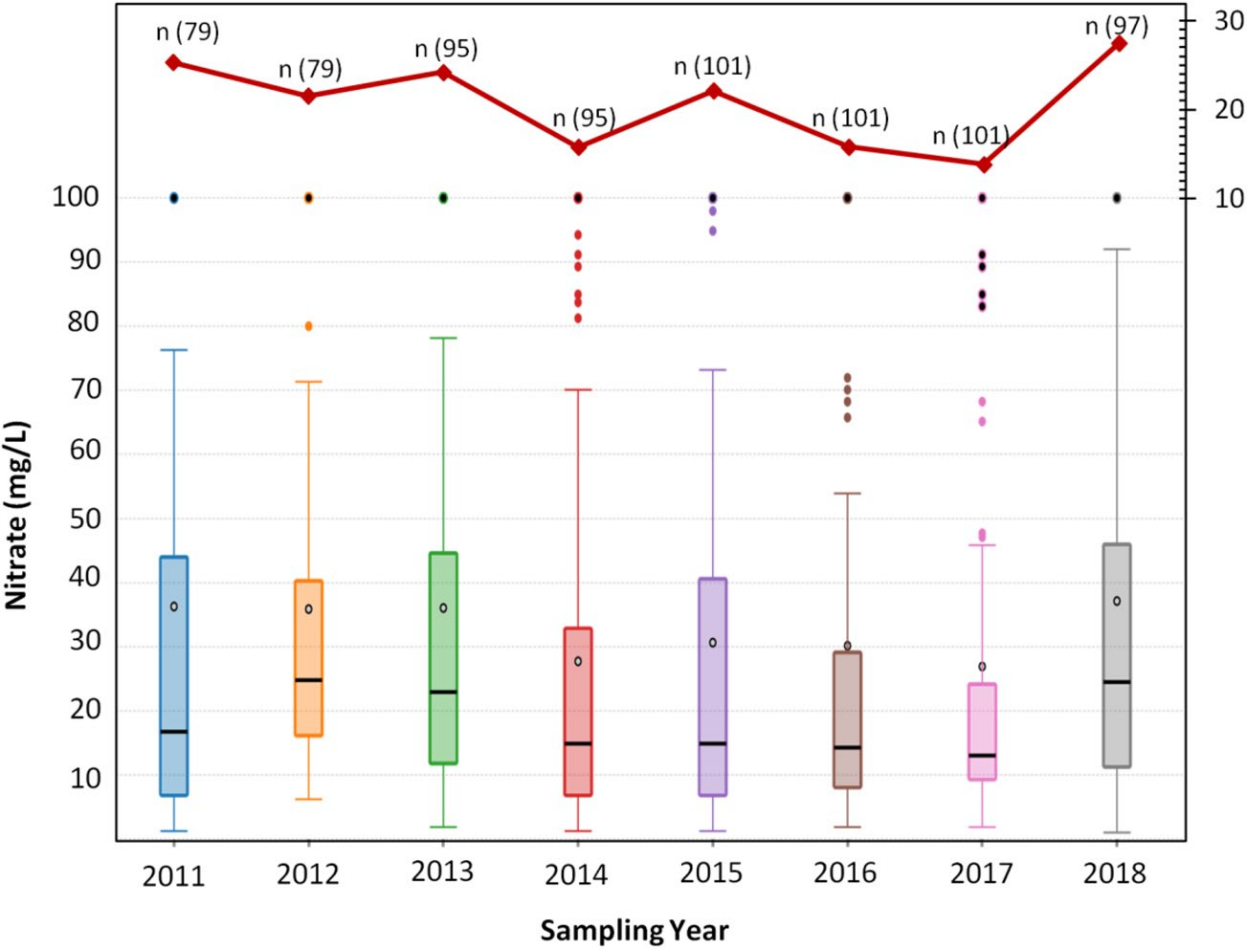
Showing the temporal changes of nitrate concentrations during 8 years (2011–2018) in groundwater of the study area. The red lines above the Box and Whisker plots illustrate the percentage of samples beyond the Indian drinking water standards (45 mg/L) and 'n' denoted as the number of groundwater samples.

**Figure 4:**
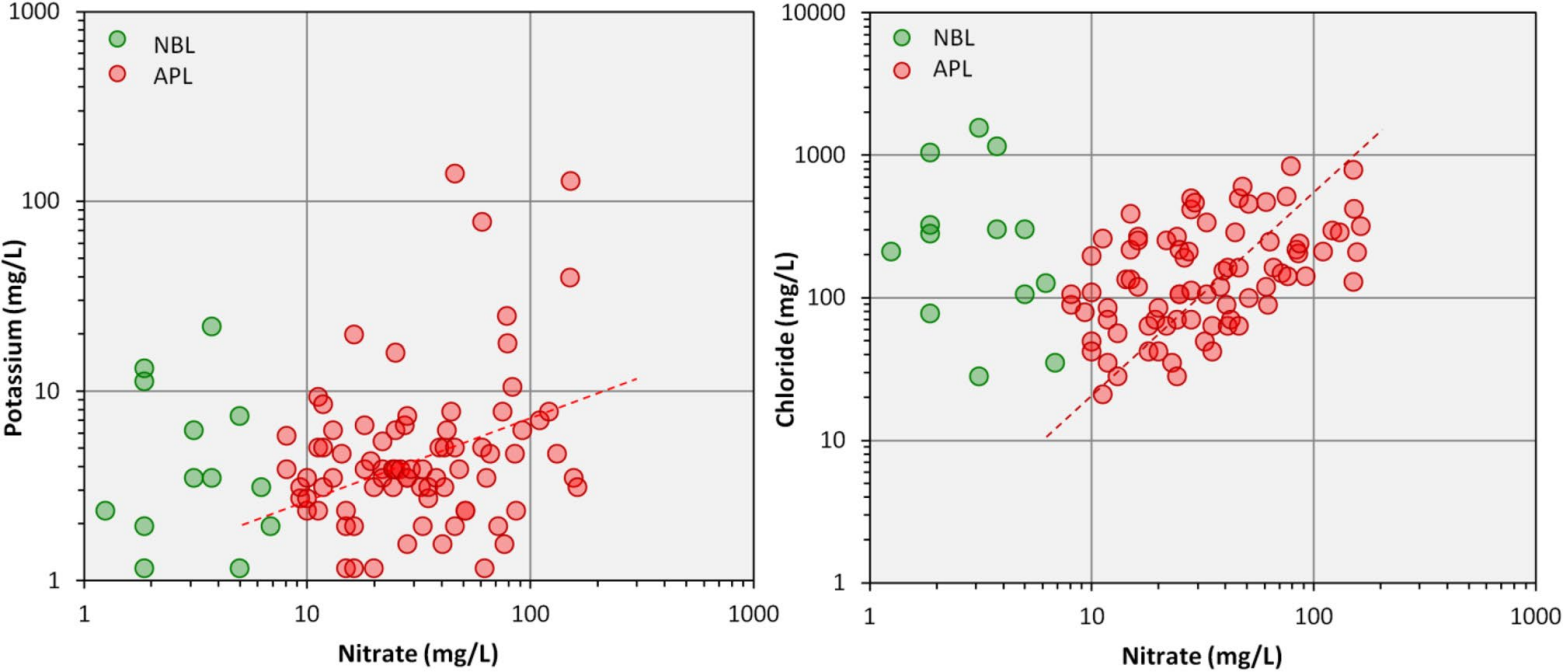
Scatter plots showing the relationship between (**a**) nitrate and potassium, and (**b**) nitrate and chloride.

The box plots^67^ in Fig. 3, demonstrated the chronological change (in time series) in nitrate concentrations in Dausa's groundwater over the past 8 years (2011 to 2018). The study area does not display any distinct temporal shift in nitrate concentrations except in 2018, where the highest groundwater sample exceeded the BIS drinking limit was about 28%. Usually, bivariate plots, NO_3_^−^ versus K^+^, and NO_3_^−^ versus Cl^−^ are commonly used to classify the possible causes of nitrate contamination^3,68–70^. Scatter plots of separate cluster including natural background level (NBL) and anthropogenic level (APL) is shown in Fig. 4. The insignificant correlation between NO_3_^−^ and K^+^ (Fig. 4a) indicated that elevated nitrate might derive from the non-point sources (including fertilizers, manure, and sewage), which cause anthropogenic pollution above the background concentration. In the APL cluster a good correlation between NO_3_^−^ and Cl^−^ was noted in the study area (Fig. 4b). It also indicated that groundwater nitrate was may be due to the sources of animal and human waste from these ions. A researcher^71^ claimed that an increase in chloride concentration with an increase in nitrate was mainly due to the septic tank.

**Figure 5:**
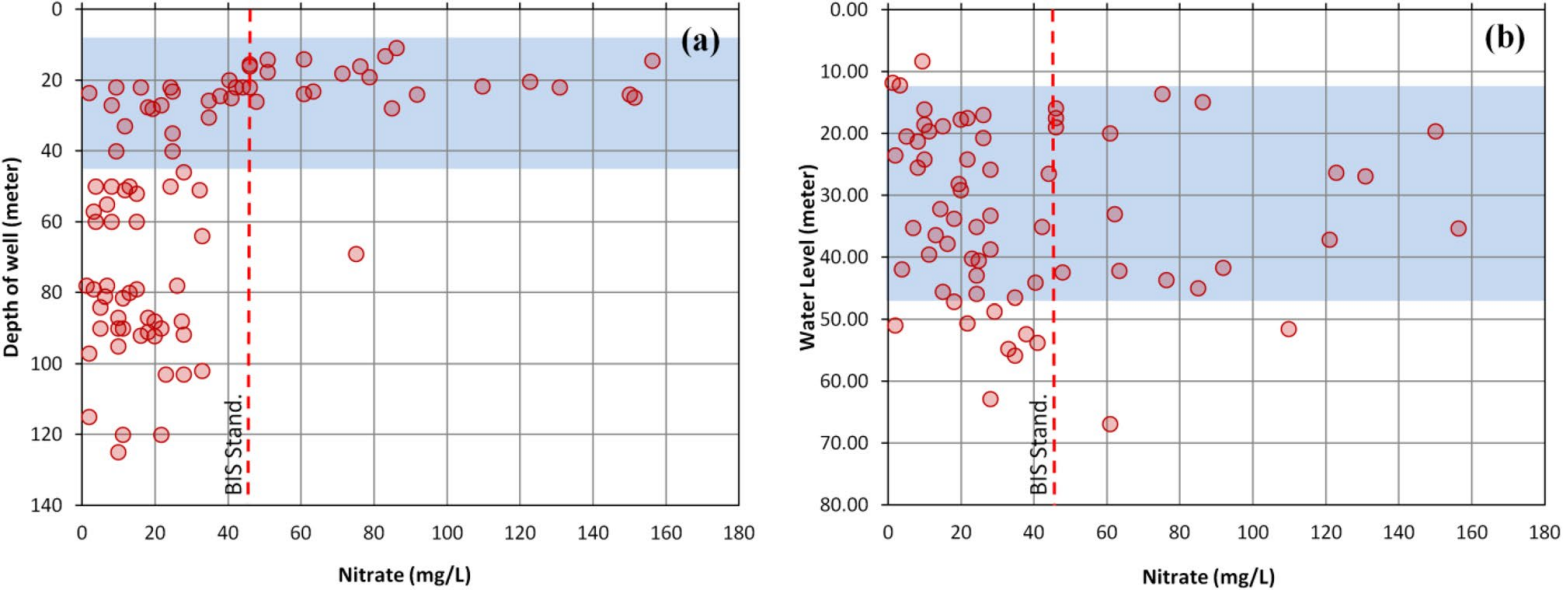
Showing (**a**) nitrate versus depth of the well, and (**b**) nitrate versus water level in the study area.

Nitrate concentrations in aquifers are usually found to decrease with depth or down gradient^26,72^. In the study area groundwater is being extracted mainly from unconfined and semi-confined aquifers. The depth of wells ranges from 11 to 125 m below ground level including both type of wells dug wells and tube wells. The high concentration of NO_3_^−^ were notice in the shallow wells which were highly influenced by nitrate fertilisers as compare to deeper wells in the study area (Fig. 5a). The water level of the study area ranged from 8 to 67 m (bgl) with an average of 33 m (bgl) and high level of NO_3_^−^ were associated with the range of water level of 11 to 47 m, bgl (Fig. 5b).

In general, principal component analysis (PCA) is widely utilized to describe the relationship between parameters of water quality and their probable source determination. In this analysis, three principal components (PCs) were generated by transformation of hydrochemical parameters including pH, TDS, K^+^, Na^+^, Mg^2+^, Ca^2+^, Cl^−^, SO_4_^2−^ , HCO_3_^−^ and NO_3_^−^) adopting Varimax orthogonal rotation and Kaiser normalisation. These PCs were examined with eigenvalues greater than one (Fig. S2) and their total variance at 49.578%, 18.916%, and 11.076%, respectively, with the cumulative variance of 79.57% (Table 2). The high and moderate positive loadings of EC (0.986), TDS (0.983), Na^+^ (0.945), Cl^−^ (0.958) and SO_4_^2−^ (0.945) were observed with PC1. This suggests that high NO_3_^−^ in the groundwater may derive from several anthropogenic sources, including fertilisers, septic tanks, domestic sewage, animal waste, and wastewater^65^. The PC3 show high positive loadings of K^+^ (0.712) and NO_3_^−^ (0.870), signifying that the source of nitrate in the groundwater was not the same as other ions. This indicates the source of elevated nitrate may come from anthropogenic activities but not from river waters^65^. The study area is dominated by agricultural land, followed by build-up areas. This scenario of the study area itself justifies that the possible source of groundwater nitrate was agricultural inputs or domestic sewage.

**Table 2:** Significant principal components (PCs) loading for hydrochemical parameters in the study area.

Component	Initial eigenvalues			Component			
	Total	% of Variance	Cumulative %	Parameters	PC1	PC2	PC3
1	5.454	49.578	49.578	pH	−0.150	**0.697**	−0.209
2	2.081	18.916	68.494	EC	**0.986**	0.121	0.095
3	1.218	11.076	79.570	TDS	**0.983**	0.119	0.120
4	0.709	6.446	86.015	Na^+^	**0.945**	0.286	0.029
5	0.671	6.102	92.117	K^+^	0.213	−0.170	**0.712**
6	0.541	4.920	97.037	Ca^2+^	−0.017	−0.708	−0.017
7	0.227	2.062	99.099	Mg^2+^	0.747	−0.412	0.180
8	0.093	0.846	99.945	Cl^−^	**0.958**	−0.193	0.041
9	0.005	0.049	99.994	SO_4_^2−^	**0.945**	0.030	0.078
10	0.001	0.005	99.999	HCO_3_^−^	0.272	**0.835**	0.074
11	0.000	0.001	100.000	NO_3_^−^	−0.023	0.067	**0.870**

Bold numbers indicate highly positive PC loadings of one parameter.

### Health risk assessment (Non-carcinogenic)

**Figure 6:**
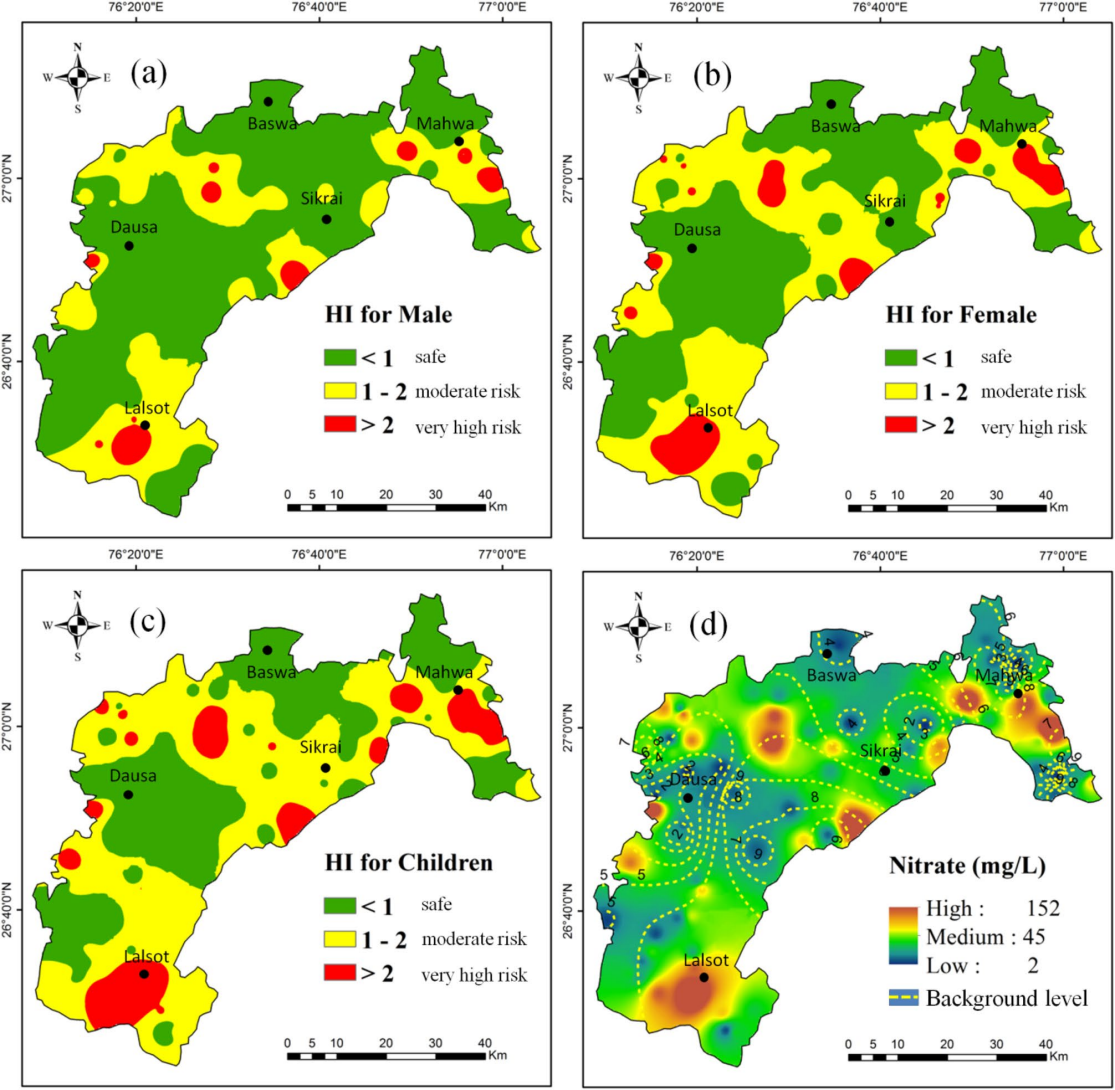
Showing spatial distribution of non-carcinogenic health risks for (**a**) Hazard Index (HI) of males, (**b**) Hazard Index (HI) of females, (**c**) Hazard Index (HI) of children in the study area, and (**d**) spatial distribution of nitrate concentration and its background concentrations with the contours. (ArcGIS Desktop. 10.3. ESRI, California, US. https://desktop.arcgis.com).

Health risk assessment including males, females, and children had been done based on the guidelines of the United State Environmental Protection Agency^33,51^, and the calculated results are shown in Table S2. Health risk (Hazard Index-HI) spatial distribution maps for males, females and children were also produced based on calculated total Hazard Index (HI_Total_) and are shown in Fig. 6. For three different age classes (12 years for children, 67 years for females, and 64 years for males^23,25^, the findings of HQ_Dermal_ were slightly lower than zero, while HQ_Oral_ ranged from 0.029 to 3.889 with an average of 0.893 for males, 0.035 to 4.597 with an average of 1.055 for females, and 0.040 to 5.259 with an average of 1.207 for children (Table 3). The results show that the exposure of nitrate directly due to drinking water ingestion was higher than the exposure due to dermal interactions in the study area. The reasonable limit for non-carcinogenic health risk is ≤ 1 (HI ≤ 1), based on the USEPA health risk standards. If the hazard index (HI) value is > 1, then the possibility of an adverse risk to human health is very high^51^. HI_Total_ values in the study area are varied from 0.029 to 3.900 (average: 0.895) for males, 0.035 to 4.609 (average: 1.058) for females and 0.040 to 5.291 (average: 1.214) for children (Table 3). Out of 97 groundwater samples, 29 samples for males, 34 samples for females, and 39 samples for infants, the nitrate exposure levels in drinking water were found to expose these age groups to serious nitrate problems. More importantly, the findings appear to suggest that children are more vulnerable to non-carcinogenic effects in the study region owing to the intake of higher nitrate concentrations in drinking water. Many other scholars have found that due to lower body weight and personality characteristics, children are more vulnerable to chronic non-carcinogenic threats than adults^3,73–77^.

**Table 3:** Summary of the estimated non-carcinogenic risks of Nitrate ingestion of drinking water and dermal exposure.

Statistics	HQ_Oral_			HQ_Dermal_			HI_Total_		
	Males	Females	Children	Males	Females	Children	Males	Females	Children
Minimum	0.029	0.035	0.040	0.000	0.000	0.000	0.029	0.035	0.040
Maximum	3.889	4.597	5.259	0.010	0.012	0.032	3.900	4.609	5.291
Average	0.893	1.055	1.207	0.002	0.003	0.008	0.895	1.058	1.214

The non-carcinogenic hazard index (HI) map (as shown in Fig. 6) clearly indicates that the northern, central, and southern parts of the study area have higher health risk zones for males, females, and children. Especially it can be noticed that the area of health risk due to groundwater nitrate is high for children as compared to the adults (Fig. 6c). As is visible from Fig. 6a, b, c, there is no detrimental impact of a non-carcinogenic risk on the visible green colour in the spatial distribution of hazard index in the study area, although the yellow and red regions show that population in these areas pose potential health threats in the study area. It indicates that the areas of yellow and red zones were in danger due to higher HI values; these areas were not recommended for direct intake of drinking water. The spatial distribution of the background values of nitrate (Fig. 6d) showed that the health hazard was high at the place where the significant difference between NBL and total concentrations of groundwater nitrate was noticed. Excessive nitrate intake through groundwater can result in adverse biological problems, such as methemoglobinemia, which is also known as blue baby syndrome, causes infant mortality, hypertension, thyroid disorders, goiter, hives, severe cyanosis, cytogenetic defects, congenital malformations, and headaches^49,65,78–82^. However, in many parts of the globe where a significant populace depends entirely on groundwater resources for drinking without pre-examination of safety problems, the non-carcinogenic health risk of NO_3_^−^ in drinking water becomes a serious issue^18,65,78,79,81,82^. In northern India, a detailed study done^74^ and assessed the human health risk of nitrate. Their research found that about 36% of samples demonstrated greater non-carcinogenic health issues on children due to higher content of nitrate in groundwater, signifying a tremendous risk to human health . Similarly, in southern India, a researcher^3^ had analysed the issues due to groundwater nitrate. Their findings reveal that about 60%, 57%, and 50% of groundwater samples were in the range of potential health risk for children, females, and males, respectively. Likewise, another study related to nitrate health implications was carried^83^ in the northern Shandong Peninsula of China, and found that about 87.6% of water samples were unfit for nitrate concentration-based consumption. The findings of their analysis also indicate that health hazards are more dangerous to infants and children due to nitrate ingestion via oral and dermal exposure pathways. Thus, owing to the drinking of contaminated groundwater, the intensity of health risk steadily increases.

### Recommendation for drinking water resource management

Both natural and anthropogenic mechanisms regulated the consistency of groundwater during the experimental period. In certain regions, because of abnormal natural environments and long-term anthropogenic factors, as we noticed in semi arid area (Dausa district, Rajasthan, India), the concentration of contaminants, especially nitrate, could be increased from other regions. In general, groundwater contamination has only been measured by taking into account the drinking water standards for the entire region or the whole country. The consideration of NBL in health risk assessment will be a crucial parameter for a better understanding of geogenic and anthropogenic contamination of any chemical parameter in the groundwater environment. However, as a result of the current study, it is indeed recommended that the local government urgently takes the requisite steps to reduce groundwater nitrate pollution and also ensures the availability of clean drinking water in the affected areas of the study area.

## Conclusions

We have analysed hydrochemical data to assess the background concentration of nitrate and possible health risks due to the elevated concentration in the drinking water in a semi arid area of Dausa district, Rajasthan, India. The natural background concentration (NBL) of groundwater nitrate is estimated using statistical approach. The estimated NBL of nitrate is 7.2 mg/L; above this background concentration, anthropogenically added nitrate is responsible for the high concentration in groundwater. The main sources of nitrate in groundwater with concentrations above background concentrations are agricultural fertilisers, and human and animal wastes. In this analysis, principal components (PCs) are generated by the processing hydrochemical parameters using Varimax orthogonal rotation and Kaiser Normalisation. The high and moderate positive loadings of EC, TDS, Na^+^, Cl^−^ and SO_4_^2−^ are observed with the PC1. This suggests that high NO_3_^−^ in the groundwater may derive from several anthropogenic sources.

Health risk assessment indicates that the oral exposure of nitrate was very high as compare to dermal contact. For the non-carcinogenic risk, our findings reveal that about 40.2%, 35.1%, and 29.9% of the groundwater samples were in the range of potential health risks for children, females, and males, respectively. The result has clearly indicated that children in the study area are more vulnerable to health hazards than women and men. The spatial distribution of nitrate background values has also revealed that the health hazard is high at the places where the significant difference between NBL and total nitrate concentrations in groundwater has been noticed. Thus the consideration of NBL in health risk assessment will be a crucial parameter for a better understanding of geogenic and anthropogenic contaminations of any chemical parameter in the groundwater system. Further the dynamic changes of NO_3_^−^ content in groundwater need to be closely monitored in order to be able to control the future spread of the pollutants. This task may be achieved by sub-surface barriers which may be physical and hydraulic barriers designed to prevent or to control the polluted groundwater flow into the desired location.

## Supplementary Information

Supplementary Information
