# Supplementary material for: Anthropogenic nitrate in groundwater and its health risks in the view of background concentration in a semi arid area of Rajasthan, India
Source: Sci Rep. 2021 Apr 29;11:9279. doi: 10.1038/s41598-021-88600-1 (PMC8085200; doi:10.1038/s41598-021-88600-1)
Supplement: Supplementary file 1 — Supplementary Information [file 41598_2021_88600_MOESM1_ESM.docx]

**Anthropogenic nitrate in groundwater and its health risks in the view of background concentration in a semi arid area of Rajasthan, India**

**Abdur Rahman^a,b*^, N.C. Mondal^a,b^ and K.K. Tiwari^c^**

^a^Earth Process Modeling Group, CSIR-National Geophysical Research Institute, Hyderabad, India

^b^Academy of Scientific & Innovative Research (AcSIR), Ghaziabad - 201 002, India

^c^National Institute of Technology (NIT), Garhwal, Uttarakhand, India

^*^**Corresponding author**: alrahman@ngri.res.in

**Supplementary material**

| Population | Shapiro-Wilk | | |
| --- | --- | --- | --- |
|  | Statistic | Degree of freedom (df) | Significant value |
| NBL | 0.909 | 19 | 0.072 |
| APL | 0.891 | 19 | 0.032 |
| Original | 0.797 | 102 | 0.000 |

Table S1. Normality test of distinguished population

Table S2. Results of non-carcinogenic health risk through drinking water intake and dermal contact.

| Sample | HQ _Oral_ | | | HQ _Dermal_ | | | HI _Total_ | | |
| --- | --- | --- | --- | --- | --- | --- | --- | --- | --- |
|  | Male | Female | Children | Male | Female | Children | Male | Female | Children |
| W-1 | 0.089 | 0.106 | 0.121 | 0.000 | 0.000 | 0.001 | 0.090 | 0.106 | 0.122 |
| W-2 | 0.581 | 0.687 | 0.786 | 0.002 | 0.002 | 0.005 | 0.583 | 0.689 | 0.791 |
| W-3 | 0.358 | 0.423 | 0.484 | 0.001 | 0.001 | 0.003 | 0.359 | 0.424 | 0.487 |
| W-4 | 0.045 | 0.053 | 0.060 | 0.000 | 0.000 | 0.000 | 0.045 | 0.053 | 0.061 |
| W-5 | 0.909 | 1.074 | 1.229 | 0.002 | 0.003 | 0.008 | 0.912 | 1.077 | 1.237 |
| W-6 | 0.462 | 0.546 | 0.625 | 0.001 | 0.001 | 0.004 | 0.463 | 0.547 | 0.628 |
| W-7 | 2.638 | 3.118 | 3.567 | 0.007 | 0.008 | 0.022 | 2.645 | 3.126 | 3.588 |
| W-8 | 0.596 | 0.705 | 0.806 | 0.002 | 0.002 | 0.005 | 0.598 | 0.706 | 0.811 |
| W-9 | 0.596 | 0.705 | 0.806 | 0.002 | 0.002 | 0.005 | 0.598 | 0.706 | 0.811 |
| W-10 | 0.089 | 0.106 | 0.121 | 0.000 | 0.000 | 0.001 | 0.090 | 0.106 | 0.122 |
| W-11 | 0.671 | 0.793 | 0.907 | 0.002 | 0.002 | 0.006 | 0.672 | 0.795 | 0.912 |
| W-12 | 0.656 | 0.775 | 0.887 | 0.002 | 0.002 | 0.005 | 0.658 | 0.777 | 0.892 |
| W-13 | 0.388 | 0.458 | 0.524 | 0.001 | 0.001 | 0.003 | 0.389 | 0.459 | 0.527 |
| W-14 | 0.343 | 0.405 | 0.463 | 0.001 | 0.001 | 0.003 | 0.344 | 0.406 | 0.466 |
| W-15 | 0.790 | 0.934 | 1.068 | 0.002 | 0.002 | 0.007 | 0.792 | 0.936 | 1.075 |
| W-16 | 1.013 | 1.198 | 1.370 | 0.003 | 0.003 | 0.008 | 1.016 | 1.201 | 1.379 |
| W-17 | 3.756 | 4.439 | 5.078 | 0.010 | 0.012 | 0.031 | 3.766 | 4.450 | 5.109 |
| W-18 | 1.997 | 2.360 | 2.700 | 0.005 | 0.006 | 0.017 | 2.002 | 2.366 | 2.717 |
| W-19 | 1.103 | 1.303 | 1.491 | 0.003 | 0.003 | 0.009 | 1.106 | 1.307 | 1.500 |
| W-20 | 1.058 | 1.251 | 1.431 | 0.003 | 0.003 | 0.009 | 1.061 | 1.254 | 1.439 |
| W-21 | 2.206 | 2.607 | 2.982 | 0.006 | 0.007 | 0.018 | 2.212 | 2.614 | 3.001 |
| W-22 | 0.388 | 0.458 | 0.524 | 0.001 | 0.001 | 0.003 | 0.389 | 0.459 | 0.527 |
| W-23 | 0.477 | 0.564 | 0.645 | 0.001 | 0.001 | 0.004 | 0.478 | 0.565 | 0.649 |
| W-24 | 0.358 | 0.423 | 0.484 | 0.001 | 0.001 | 0.003 | 0.359 | 0.424 | 0.487 |
| W-25 | 1.878 | 2.219 | 2.539 | 0.005 | 0.006 | 0.016 | 1.883 | 2.225 | 2.555 |
| W-26 | 0.119 | 0.141 | 0.161 | 0.000 | 0.000 | 0.001 | 0.120 | 0.141 | 0.162 |
| W-27 | 0.313 | 0.370 | 0.423 | 0.001 | 0.001 | 0.003 | 0.314 | 0.371 | 0.426 |
| W-28 | 1.803 | 2.131 | 2.438 | 0.005 | 0.006 | 0.015 | 1.808 | 2.137 | 2.453 |
| W-29 | 0.075 | 0.088 | 0.101 | 0.000 | 0.000 | 0.001 | 0.075 | 0.088 | 0.101 |
| W-30 | 0.581 | 0.687 | 0.786 | 0.002 | 0.002 | 0.005 | 0.583 | 0.689 | 0.791 |
| W-31 | 0.045 | 0.053 | 0.060 | 0.000 | 0.000 | 0.000 | 0.045 | 0.053 | 0.061 |
| W-32 | 0.238 | 0.282 | 0.322 | 0.001 | 0.001 | 0.002 | 0.239 | 0.283 | 0.324 |
| W-33 | 0.522 | 0.616 | 0.705 | 0.001 | 0.002 | 0.004 | 0.523 | 0.618 | 0.710 |
| W-34 | 0.045 | 0.053 | 0.060 | 0.000 | 0.000 | 0.000 | 0.045 | 0.053 | 0.061 |
| W-35 | 0.268 | 0.317 | 0.363 | 0.001 | 0.001 | 0.002 | 0.269 | 0.318 | 0.365 |
| W-36 | 0.835 | 0.986 | 1.128 | 0.002 | 0.003 | 0.007 | 0.837 | 0.989 | 1.135 |
| W-37 | 1.222 | 1.444 | 1.652 | 0.003 | 0.004 | 0.010 | 1.225 | 1.448 | 1.662 |
| W-38 | 0.164 | 0.194 | 0.222 | 0.000 | 0.001 | 0.001 | 0.164 | 0.194 | 0.223 |
| W-39 | 0.313 | 0.370 | 0.423 | 0.001 | 0.001 | 0.003 | 0.314 | 0.371 | 0.426 |
| W-40 | 0.969 | 1.145 | 1.310 | 0.003 | 0.003 | 0.008 | 0.971 | 1.148 | 1.318 |
| W-41 | 0.835 | 0.986 | 1.128 | 0.002 | 0.003 | 0.007 | 0.837 | 0.989 | 1.135 |
| W-42 | 0.119 | 0.141 | 0.161 | 0.000 | 0.000 | 0.001 | 0.120 | 0.141 | 0.162 |
| W-43 | 1.520 | 1.797 | 2.055 | 0.004 | 0.005 | 0.013 | 1.524 | 1.801 | 2.068 |
| W-44 | 0.596 | 0.705 | 0.806 | 0.002 | 0.002 | 0.005 | 0.598 | 0.706 | 0.811 |
| W-45 | 1.103 | 1.303 | 1.491 | 0.003 | 0.003 | 0.009 | 1.106 | 1.307 | 1.500 |
| W-46 | 2.072 | 2.448 | 2.801 | 0.006 | 0.007 | 0.017 | 2.077 | 2.455 | 2.818 |
| W-47 | 2.042 | 2.413 | 2.761 | 0.005 | 0.006 | 0.017 | 2.047 | 2.419 | 2.778 |
| W-48 | 0.775 | 0.916 | 1.048 | 0.002 | 0.002 | 0.006 | 0.777 | 0.918 | 1.054 |
| W-49 | 0.149 | 0.176 | 0.202 | 0.000 | 0.000 | 0.001 | 0.149 | 0.177 | 0.203 |
| W-50 | 0.477 | 0.564 | 0.645 | 0.001 | 0.001 | 0.004 | 0.478 | 0.565 | 0.649 |
| W-51 | 0.522 | 0.616 | 0.705 | 0.001 | 0.002 | 0.004 | 0.523 | 0.618 | 0.710 |
| W-52 | 0.671 | 0.793 | 0.907 | 0.002 | 0.002 | 0.006 | 0.672 | 0.795 | 0.912 |
| W-53 | 1.490 | 1.761 | 2.015 | 0.004 | 0.005 | 0.012 | 1.494 | 1.766 | 2.027 |
| W-54 | 0.283 | 0.335 | 0.383 | 0.001 | 0.001 | 0.002 | 0.284 | 0.336 | 0.385 |
| W-55 | 0.626 | 0.740 | 0.846 | 0.002 | 0.002 | 0.005 | 0.628 | 0.742 | 0.852 |
| W-56 | 0.030 | 0.035 | 0.040 | 0.000 | 0.000 | 0.000 | 0.030 | 0.035 | 0.041 |
| W-57 | 1.893 | 2.237 | 2.559 | 0.005 | 0.006 | 0.016 | 1.898 | 2.243 | 2.575 |
| W-58 | 3.145 | 3.716 | 4.252 | 0.008 | 0.010 | 0.026 | 3.153 | 3.726 | 4.278 |
| W-59 | 0.268 | 0.317 | 0.363 | 0.001 | 0.001 | 0.002 | 0.269 | 0.318 | 0.365 |
| W-60 | 0.238 | 0.282 | 0.322 | 0.001 | 0.001 | 0.002 | 0.239 | 0.283 | 0.324 |
| W-61 | 0.984 | 1.163 | 1.330 | 0.003 | 0.003 | 0.008 | 0.986 | 1.166 | 1.338 |
| W-62 | 2.906 | 3.435 | 3.929 | 0.008 | 0.009 | 0.024 | 2.914 | 3.444 | 3.953 |
| W-63 | 1.461 | 1.726 | 1.975 | 0.004 | 0.005 | 0.012 | 1.464 | 1.731 | 1.987 |
| W-64 | 0.268 | 0.317 | 0.363 | 0.001 | 0.001 | 0.002 | 0.269 | 0.318 | 0.365 |
| W-65 | 1.103 | 1.303 | 1.491 | 0.003 | 0.003 | 0.009 | 1.106 | 1.307 | 1.500 |
| W-66 | 0.388 | 0.458 | 0.524 | 0.001 | 0.001 | 0.003 | 0.389 | 0.459 | 0.527 |
| W-67 | 0.224 | 0.264 | 0.302 | 0.001 | 0.001 | 0.002 | 0.224 | 0.265 | 0.304 |
| W-68 | 0.075 | 0.088 | 0.101 | 0.000 | 0.000 | 0.001 | 0.075 | 0.088 | 0.101 |
| W-69 | 0.432 | 0.511 | 0.584 | 0.001 | 0.001 | 0.004 | 0.433 | 0.512 | 0.588 |
| W-70 | 0.671 | 0.793 | 0.907 | 0.002 | 0.002 | 0.006 | 0.672 | 0.795 | 0.912 |
| W-71 | 0.581 | 0.687 | 0.786 | 0.002 | 0.002 | 0.005 | 0.583 | 0.689 | 0.791 |
| W-72 | 0.283 | 0.335 | 0.383 | 0.001 | 0.001 | 0.002 | 0.284 | 0.336 | 0.385 |
| W-73 | 3.890 | 4.597 | 5.259 | 0.010 | 0.012 | 0.032 | 3.900 | 4.609 | 5.292 |
| W-74 | 0.045 | 0.053 | 0.060 | 0.000 | 0.000 | 0.000 | 0.045 | 0.053 | 0.061 |
| W-75 | 0.432 | 0.511 | 0.584 | 0.001 | 0.001 | 0.004 | 0.433 | 0.512 | 0.588 |
| W-76 | 0.224 | 0.264 | 0.302 | 0.001 | 0.001 | 0.002 | 0.224 | 0.265 | 0.304 |
| W-77 | 0.238 | 0.282 | 0.322 | 0.001 | 0.001 | 0.002 | 0.239 | 0.283 | 0.324 |
| W-78 | 3.637 | 4.298 | 4.917 | 0.010 | 0.011 | 0.030 | 3.646 | 4.309 | 4.947 |
| W-79 | 0.522 | 0.616 | 0.705 | 0.001 | 0.002 | 0.004 | 0.523 | 0.618 | 0.710 |
| W-80 | 0.194 | 0.229 | 0.262 | 0.001 | 0.001 | 0.002 | 0.194 | 0.230 | 0.264 |
| W-81 | 0.671 | 0.793 | 0.907 | 0.002 | 0.002 | 0.006 | 0.672 | 0.795 | 0.912 |
| W-82 | 0.551 | 0.652 | 0.746 | 0.001 | 0.002 | 0.005 | 0.553 | 0.653 | 0.750 |
| W-83 | 0.194 | 0.229 | 0.262 | 0.001 | 0.001 | 0.002 | 0.194 | 0.230 | 0.264 |
| W-84 | 1.148 | 1.356 | 1.552 | 0.003 | 0.004 | 0.010 | 1.151 | 1.360 | 1.561 |
| W-85 | 0.939 | 1.110 | 1.269 | 0.002 | 0.003 | 0.008 | 0.941 | 1.113 | 1.277 |
| W-86 | 0.984 | 1.163 | 1.330 | 0.003 | 0.003 | 0.008 | 0.986 | 1.166 | 1.338 |
| W-87 | 1.833 | 2.166 | 2.478 | 0.005 | 0.006 | 0.015 | 1.838 | 2.172 | 2.494 |
| W-88 | 1.222 | 1.444 | 1.652 | 0.003 | 0.004 | 0.010 | 1.225 | 1.448 | 1.662 |
| W-89 | 0.700 | 0.828 | 0.947 | 0.002 | 0.002 | 0.006 | 0.702 | 0.830 | 0.953 |
| W-90 | 1.461 | 1.726 | 1.975 | 0.004 | 0.005 | 0.012 | 1.464 | 1.731 | 1.987 |
| W-91 | 1.580 | 1.867 | 2.136 | 0.004 | 0.005 | 0.013 | 1.584 | 1.872 | 2.149 |
| W-92 | 0.790 | 0.934 | 1.068 | 0.002 | 0.002 | 0.007 | 0.792 | 0.936 | 1.075 |
| W-93 | 1.714 | 2.026 | 2.317 | 0.005 | 0.005 | 0.014 | 1.718 | 2.031 | 2.332 |
| W-94 | 0.238 | 0.282 | 0.322 | 0.001 | 0.001 | 0.002 | 0.239 | 0.283 | 0.324 |
| W-95 | 0.283 | 0.335 | 0.383 | 0.001 | 0.001 | 0.002 | 0.284 | 0.336 | 0.385 |
| W-96 | 3.607 | 4.263 | 4.876 | 0.010 | 0.011 | 0.030 | 3.616 | 4.274 | 4.906 |
| W-97 | 0.358 | 0.423 | 0.484 | 0.001 | 0.001 | 0.003 | 0.359 | 0.424 | 0.487 |


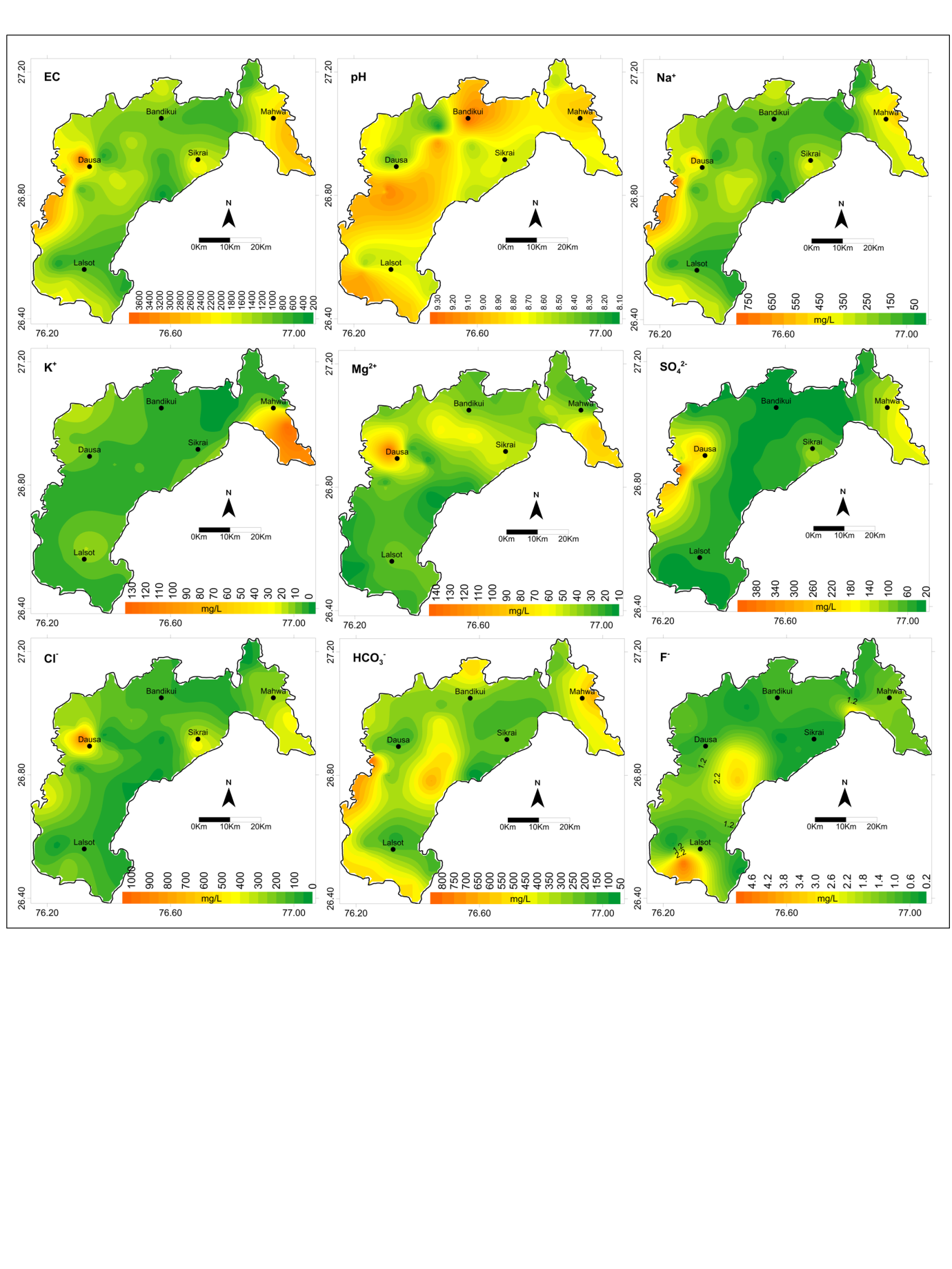
Figure S1. Spatial distribution of hydrochemical parameters in a semi-arid area, Rajasthan, India. (Surfer 13, https://www.goldensoftware.com)


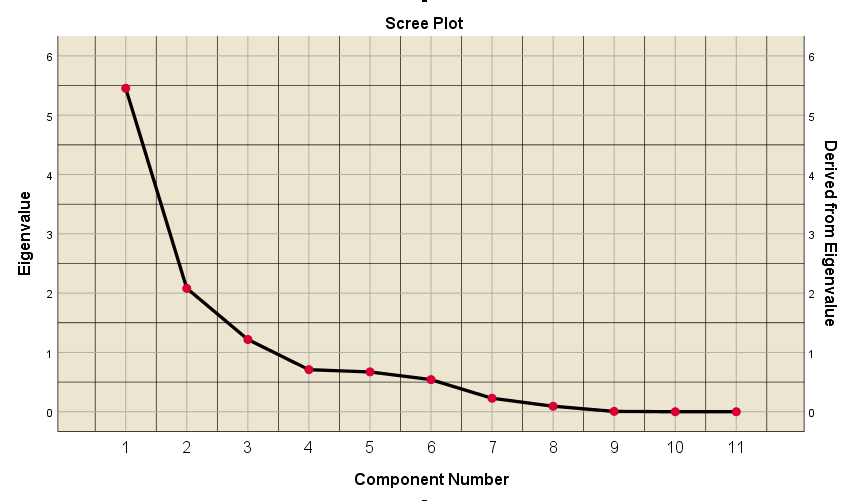
 Figure S2. Scree plot for principal component analysis showing 3 components with >1 Eigenvalues value (IBM SPSS 25.0, https://www.ibm.com)
